# Current landscape for the management of facioscapulohumeral muscular dystrophy and emerging treatment modalities: A literature review

**DOI:** 10.3934/Neuroscience.2025016

**Published:** 2025-06-25

**Authors:** Ubaid Ansari, Dawnica Nadora, Lauren Ong, Romteen Sedighi, Ethan Tabaie, Zaid Ansari, Meraj Alam, Burhaan Syed, Noorhan Amani, Sarah Preiss-Farzanegan

**Affiliations:** California Northstate University College of Medicine, USA

**Keywords:** facioscapulohumeral muscular dystrophy, neuromuscular disorder, muscle weakness, pharmacological treatments, gene therapy, rehabilitation strategies, exercise interventions

## Abstract

Facioscapulohumeral Muscular Dystrophy (FSHD) is a genetic disorder characterized by progressive muscle weakness, primarily affecting the facial, shoulder, and upper arm muscles. In this literature review, we examined the available treatments for FSHD, covering established methods and experimental approaches. We began with an overview of pharmacological treatments, emphasizing the importance of physical therapy and rehabilitation in maintaining muscle strength, improving mobility, preventing contractures, and respiratory therapy for severe cases. We also explored exercise interventions, addressing the debate surrounding exercise in FSHD patients, and highlight the possible benefits of aerobic and strength training, as well as ongoing research into safe exercise protocols. Additionally, the use of assistive devices and orthotics, such as braces and mobility aids, is discussed, along with surgical interventions like scapular fixation surgery and corrective procedures for foot drop. Emerging therapeutic strategies, including gene therapy focusing on DUX4 silencing and CRISPR-Cas9 technology, were evaluated. The potential of antisense oligonucleotides and myostatin inhibitors was reviewed, along with the challenges and ethical considerations associated with cell-based therapies. We aimed to inform researchers and advance treatment strategies for FSHD patients.

## 1. Introduction

Facioscapulohumeral muscular dystrophy (FSHD) is a genetically inherited neuromuscular disorder that follows an autosomal dominant inheritance pattern [1]. It is regarded as the third most common form of muscular dystrophy, with an estimated prevalence of 1 in 20,000 people worldwide [2]. This progressive condition predominantly affects the muscles of the face (facio), shoulders (scapulo), and upper arms (humeral), although it may also involve muscles in the legs, hips, and abdomen as the disease advances [1]. While most patients experience gradual and asymmetric muscle weakness, the severity and progression can vary widely. In some individuals, FSHD manifests early in life and progresses rapidly, while others may remain relatively unaffected until late adulthood. In addition to muscle weakness, non-muscle symptoms such as retinal vascular abnormalities, hearing loss, and chronic pain are reported in some cases [1]. Regardless of the presenting symptoms, the disease poses significant challenges to the affected individuals, impacting their quality of life, physical abilities, and functional independence.

The pathogenesis of FSHD has been closely linked to aberrant expression of the DUX4 gene, a toxic protein product that becomes inappropriately active in muscle cells due to deletions in the D4Z4 macrosatellite repeat on chromosome 4 [3]. Understanding this genetic basis has been pivotal in advancing research on FSHD, particularly in the exploration of potential gene therapies. However, despite these advances, the therapeutic landscape for FSHD needs to be developed, with no approved disease-modifying treatments currently available. Management of FSHD is often multidisciplinary, focusing on symptomatic relief, functional maintenance, and slowing the progression of disability [4]. This disease's progressive nature and variability in severity pose unique emotional and social challenges for patients and their families, emphasizing the need for comprehensive care approaches. FSHD diagnosis is established primarily through molecular genetic testing, which reveals two subtypes of FSHD. FSHD1, which accounts for about 95% of cases, is caused by heterozygous pathogenic contraction of the D4Z4 repeat array on chromosome 4q35 permissive haplotype [1]. On the other hand, FSHD2, which accounts for the other 5% of cases, is caused by hypomethylation of the D4Z4 repeat array on chromosome 4q35 permissive haplotype, because of either a heterozygous SMCHD1 mutation or a heterozygous DNMT3B mutation [1].

Here, we seek to comprehensively examine the current treatment options available for FSHD, evaluating both established and experimental approaches. One of the core aspects of FSHD management involves physical therapy, which plays a critical role in preserving muscle strength and preventing contractures [5]. Though once controversial due to concerns about exacerbating muscle damage, exercise has emerged as a promising area of research, with researchers exploring its potential to enhance mobility and quality of life for patients with FSHD. Pharmacologic treatments are another key focus. Various drugs are being investigated for their ability to target the underlying pathophysiology, such as inflammation, muscle atrophy, and gene regulation [6].

Beyond conventional interventions, we also explore emerging therapies, including gene-targeted approaches and antisense oligonucleotides [7]. Advances in these fields may offer new hope for slowing or halting disease progression. In addition to these interventions, the roles of assistive devices, orthotics, and surgical options are discussed as they contribute to the holistic management of FSHD [8]. By synthesizing the latest research, we aim to provide a comprehensive resource that can guide future investigations and ultimately contribute to the development of more effective, targeted therapies for individuals living with FSHD.

### 1.1. Current landscape of FSHD treatments

#### 1.1.1. Pharmacological treatments

*Anti-inflammatory agents (e.g., corticosteroids)*. Since inflammation is typically seen in FSHD patients, corticosteroids have been investigated in the past to determine whether their use can help alleviate FSHD symptoms, similar to their use in managing Duchenne muscular dystrophy. Tiwali et al. performed a pilot, open-label trial of prednisone in eight subjects fulfilling strict diagnostic criteria for FSHD (Table 1) [9]. The participants were prescribed prednisone (1.5 mg/kg/day; maximum 80 mg/day) for 12 weeks. However, no significant changes were observed in muscle mass or strength at the end of the study period. Thus, the study suggests that alternative therapeutic approaches must be explored and that researchers should focus on more targeted treatments that address the underlying genetic and molecular mechanisms of FSHD.

*Antioxidants and supplements (e.g., vitamin C, vitamin E, CoQ10, zinc gluconate, and selenomethionine)*. Given the growing evidence that oxidative stress influences FSHD pathology, researchers evaluated antioxidant supplementation with vitamins and minerals to determine its potential in reducing free radical formation and its impact on muscle function in patients with FSHD.

A pilot randomized double-blind placebo-controlled study was conducted by Passerieux et al. to assess the effects of vitamin C, vitamin E (as alpha-tocopherol), zinc gluconate, and selenomethionine in FSHD patients [10]. Subjects were randomized to receive 500 mg of vitamin C, 400 mg of vitamin E, 25 mg of zinc gluconate, 200 µg selenomethionine (n = 26), or a matching placebo (n = 27) once a day for 17 weeks. After the study period, they reported that with oral supplementation of the antioxidant mix, the experimental group showed a significant difference in maximal voluntary contraction (MVC) and endurance limit time (Tlim) of the quadriceps compared to the placebo group. It was also noted that antioxidant levels and oxidative stress markers improved significantly with supplementation. Additionally, the supplements enhanced quadriceps function by increasing antioxidant response and reducing oxidative stress but had no significant impact on the two-minute walking test (2-MWT). The authors also emphasized the need for an exercise protocol for FSHD patients that may provide important insights into its effects on oxidative stress and inflammation markers. Given the scarcity of research, further studies are warranted to support the potential of antioxidants as a treatment option for FSHD patients.

*Drugs targeting muscle atrophy (e.g., beta-agonists)*. Βeta-2 adrenergic agonists are mostly used as a bronchodilator for asthma and other respiratory conditions and are not typically a first-line treatment for FSHD. However, there has been some interest in exploring its potential for improving muscle strength in FSHD since animal and human studies show that β2-adrenergic agonists produce anabolic effects on skeletal muscles [11]–[13]. The results of several clinical trials have been mixed, with some studies suggesting modest benefits in muscle strength, while others show minimal or no effect.

In a randomized, double-blind, placebo-controlled trial of albuterol in facioscapulohumeral dystrophy, 90 participants were randomized to three groups: placebo, 8.0 mg albuterol twice daily, or 16.0 mg albuterol twice daily [14]. After one year of treatment, no significant differences were observed between the groups in maximum voluntary isometric contraction (MVIC) scores or strength measured by manual muscle testing (MMT). However, the treatment group did show a significant increase in grip strength, p = 0.03. Additionally, the high-dose group demonstrated a significant increase in lean mass by DEXA compared to placebo (p = 0.007). Albuterol was well tolerated, and side effects that were reported included cramps, tremors, insomnia, and nervousness. In a similar study design, van der Kooi et al. reported that after supplementation of sustained-released 8 mg albuterol twice a day for 26 weeks to FSHD patients, albuterol did not improve global strength or function. However, it increased muscle mass and improved some strength measures [15]. In 2009, Payan et al. conducted a placebo-controlled, double-blind, randomized study at three neuromuscular disorder centers with 112 ambulatory patients aged 18 to 60 with genetically confirmed FSHD. The participants were divided into two groups (n = 56) and given either a sustained-release oral salbutamol dose of 16 mg daily or a placebo. The results showed no significant improvement in muscle strength or motor function with salbutamol compared to the placebo. They concluded that salbutamol should not be considered a routine treatment for FSHD, though potential anabolic effects warrant further investigation [16]. Based on the literature, the use of albuterol in FSHD is relatively rare, and further research is needed to determine its efficacy as a treatment option for FSHD.

*Experimental drug trials (e.g., losmapimod)*. Currently, there are no approved disease-modifying treatments available for FSHD. To address this unmet need, losmapimod, a small molecule that inhibits p38α MAPK and p38β MAPK, was assessed for its safety and efficacy by Tawil et al. [17]. They conducted a randomized, double-blind, placebo-controlled phase 2b trial at 17 neurology centers across Canada, France, Spain, and the United States. The study included 80 adults ages 18 to 65 with a diagnosis of FSHD1 who were randomly assigned to receive either oral losmapimod (15 mg twice daily) or a placebo for 48 weeks. The primary endpoint was assessing the change in DUX4-driven gene expression in skeletal muscle biopsies using quantitative RT-PCR at week 16 or 36. Results showed no significant difference in changes in DUX4-driven gene expression between the losmapimod and placebo groups (p = 0.56). In addition, no adverse events were reported during the study. Despite these results, a Phase 3 study for losmapimod in adults was planned due to the improvements in muscle fat infiltration, the measure of shoulder girdle function, and patient-reported global impression of change compared with placebo. However, as of September 2024, the pharmaceutical company conducting the Phase 3 REACH trial on losmapimod suspended its development since it did not demonstrate any significant change from baseline in Reachable Workspace Analysis (RSA) with losmapimod compared to the placebo treatment [18]. The study results show that those who were allocated losmapimod had a 0.013 (±0.007) improvement in RSA at week 48 compared to placebo patients who showed a 0.010 (±0.007) improvement in RWS (p-value = 0.75). As for Muscle Fat Infiltration (MFI) measured via Magnetic Resonance Imaging (MRI), subjects who received losmapimod had an increase of 0.42% in MFI at week 48 compared to participants receiving a placebo who showed an increase of 0.576% in MFI (p-value = 0.16). These results highlight the ongoing challenge of developing an effective treatment for FSHD.

Other innovative approaches, such as Avidity's emerging RNA-targeted therapies, offer new possibilities for addressing the underlying genetic mechanisms of the disease [19]. Avidity's delpacibart braxlosiran (del-brax) is the first investigational therapy designed to treat FSHD by targeting the abnormal expression of DUX4. Early findings of the phase 2 trial suggest that del-brax could significantly decrease DUX4 gene expression, as it showed an average reduction of over 50% in DUX4 expression and a 25% decrease in DUX4-related proteins after four months of treatment. The del-brax group also demonstrated improvements in muscle strength and function compared to the placebo group. In addition, after drug supplementation, creatine kinase levels were reduced, indicating a decrease in muscle damage. While these findings are preliminary, it is unclear how this drug can impact FSHD symptoms. Ongoing research is crucial to determine these preliminary results' long-term efficacy and clinical significance.

### 1.2. Physical therapy and rehabilitation

Physical therapy is a critical component of the multidisciplinary approach in the management of FSHD, often involving an initial interview to gauge the patient's functional deficits, such as activities of daily living and movement capabilities, as well as manual assessment of musculoskeletal symptoms (joint amplitude, strength, posture, gait, balance, and tone scoring). The severity of the disease course informs the frequency and rigor of future, goal-oriented physical therapy, which may include contracture-opposed stretching, assistance with active and passive muscle movement, and analgesic massages [20]. High-intensity regimens that overwork the body and cause prolonged muscle soreness should generally be avoided; instead, emphasis should be placed on strengthening the core, balance, joint flexibility, energy maintenance, and endurance through gradual activities such as sit-to-stand transfers, floor transfers, or alternative types of exercise such as yoga, Pilates, and hydrotherapy [21]. As musculoskeletal pain is often a significant symptom in FSHD, nonsteroidal anti-inflammatory medications, along with anticonvulsants or antidepressants, may be considered; however, consultation with a physical therapist is recommended to give patients a greater understanding of physiological pain mechanisms and establish a mild-moderate training program individualized to the patient's capabilities [22]. Strength-enhancing physical therapy in the treatment of FSHD and other neuromuscular disorders has demonstrated limited improvements in mean torque, strength, and endurance with repeated isotonic flexion and extension of the knee, elbow, trunk, hip, and shoulder in early research studies [23]–[25]. While recent systematic analyses have not demonstrated significant benefits from these kinds of strength-based exercise regimens broadly in FSHD, they may factor into the interdisciplinary approach of the disease and have shown success in case studies [26],[27]. As such, physical therapy remains one of the modalities to address fatigue, pain, and functional decline in patients with FSHD.

### 1.3. Exercise interventions

There has been long standing controversy regarding exercise interventions in FSHD, as concerns about exacerbating muscle damage have made clinicians cautious in prescribing physical activity. Despite these concerns, studies suggest that carefully structured exercise regimens can offer benefits, provided the interventions are designed with safety and disease progression in mind.

#### 1.3.1. Controversy over exercise in FSHD patients

Muscular dystrophy in general, including FSHD, leads to significant muscle loss and weakness, which has historically raised doubts about the safety of exercise in these populations. A systematic review and meta-analysis by Gianola et al. highlighted this uncertainty, finding no significant improvements in muscle strength for patients with FSHD who engaged in exercise, though there were modest improvements in endurance during walking [28]. The study emphasizes that while some functional gains are observed, the impact on muscle strength remains limited, underscoring the need for further research to determine optimal exercise types for these patients.

#### 1.3.2. Benefits of aerobic and strength training

While the concern over potential muscle damage persists, research supports the safety and efficacy of certain exercise protocols in FSHD patients. A six-month randomized controlled trial (RCT) by Bankolé et al. demonstrated significant improvements in aerobic capacity, muscle strength, and walking distance in patients who participated in a home-based cycling regimen that combined strength and interval training [29]. Importantly, this study revealed no worsening of the dystrophic muscle pathology, suggesting that such interventions can enhance quality of life without further muscle degradation. Similarly, another study by Olsen et al. found that a 12-week program of low-intensity aerobic exercise improved maximal oxygen uptake in FSHD patients, further supporting the safety of aerobic training for improving endurance and overall exercise performance without causing harm [30].

#### 1.3.3. Ongoing research on safe exercise protocols

Ongoing research continues to refine our understanding of the role of physical activity in FSHD management. A retrospective study by Bettio et al. found that individuals with FSHD who engaged in physical activity at a young age exhibited less severe clinical presentations of the disease later in life, suggesting that early exercise interventions may slow disease progression [31]. This finding aligns with the growing consensus that controlled physical activity can benefit FSHD patients, but the need for individualized and carefully monitored exercise protocols remains paramount. Developing such protocols will require further trials to better understand the long-term effects and identify which types of exercise provide the most benefit with the least risk.

### 1.4. Assistive devices and orthotics

Assistive devices are used to enhance mobility, improve daily function, and reduce fatigue in individuals with FHSD. Orthoses are used to support muscles affected by the disease. These include ankle-foot orthoses (AFO), floor reaction ankle-foot orthoses (FRAFO), and knee-ankle-foot orthoses (KAFO), depending on the severity of the muscle weakness [32]. An AFO is quite effective for foot drop; however, in patients with quadriceps atrophy, a simple AFO could ergonomically hinder gait due to weakened knee extension [33]. Using a FRAFO or KAFO for advanced quadriceps involvement can alleviate these ergonomic issues. Advancements in ease of application and lighter materials will make it easier for patients to use orthoses [34]. Braces or casts for winging scapula and shoulder dysfunction also exist; however, the benefits of their use are unclear [8]. Additionally, although stabilization is important, the risk of prolonged immobilization can lead to further muscle loss, dysfunction, and brachial plexus compression.

### 1.5. Surgical interventions

#### 1.5.1. Scapulothoracic arthrodesis in FSHD

For FSHD patients experiencing severe scapular winging due to progressive muscle weakness, Scapulothoracic Arthrodesis (STA) is the primary surgical treatment. STA stabilizes the scapula to improve shoulder mobility, allowing muscles like the deltoid and supraspinatus to function more efficiently during shoulder abduction and flexion. Various techniques, including multifilament cables and cerclage wires, have been used successfully, showing improved shoulder elevation and quality of life post-surgery. For instance, one study demonstrated that postoperative shoulder flexion improved from 71° to 109°, and the UCLA Shoulder Score increased from 18.4 to 27.9, indicating significant functional gains [35].

However, STA can present complications, such as pulmonary issues or wire breakage. Proper patient selection and a multidisciplinary team, including neurologists, geneticists, and orthopedic surgeons, are essential for minimizing risks. Other surgical options, like tendon transfers, address FSHD-related foot drop or facial weakness, with functional and cosmetic improvements evaluated on a case-by-case basis [32].

#### 1.5.2. Gold-weight implantation for FSHD-related lagophthalmos

FSHD-related facial muscle weakness can lead to lagophthalmos, a condition in which patients are unable to fully close their eyelids. This condition poses a risk of corneal damage due to inadequate eyelid closure and exposure to environmental irritants. Traditional treatments such as tarsorrhaphy or prosthetic devices are often associated with complications, including infection, extrusion, and unsatisfactory cosmetic outcomes.

Gold-weight implantation in the upper eyelid has emerged as a potential intervention for paralytic lagophthalmos in FSHD, offering both functional and cosmetic benefits. The weight assists in eyelid closure using gravity to compensate for weakened eyelid muscles. In a case study involving a 64-year-old woman with FSHD, gold-weight implantation successfully corrected severe lagophthalmos, resulting in complete eyelid closure, improved blinking, and enhanced ocular comfort. The procedure was also associated with significant cosmetic improvements and no major postoperative complications [36]. Given its simplicity, reversibility, and low complication rate, gold-weight implantation is a reliable early intervention for preventing corneal damage in patients with FSHD-related facial muscle weakness.

## 2. Emerging therapeutic approaches

As research advances in the field of FSHD, a variety of innovative therapeutic strategies are being explored to target the disease's complex biological mechanisms. Collectively, these emerging therapeutic approaches represent a multifaceted effort to address the underlying causes of FSHD and improve patient outcomes. Ongoing clinical trials for FSHD treatments are summarized in Table 1.

### 2.1. Gene therapy and genetic approaches

#### 2.1.1. DUX4 silencing techniques

FSHD results from the abnormal expression of DUX4, a transcription factor encoded within the D4Z4 repeat region on chromosome 4. Except for the testis and thymus, DUX4 expression is repressed in adult tissues by epigenetic silencing of the D4Z4 repeat arrays [37]. FSHD results from the loss of this epigenetic repression, either due to contraction of the D4Z4 array (FSHD type 1, 95% of patients) or mutations in genes involved in maintaining repression via structural maintenance of chromosomes, such as SMCHD1 (FSHD type 2, 5% of patients) [38],[39].

This leads to the inappropriate expression of DUX4 in muscle cells, contributing to progressive muscle degeneration.

Research has highlighted the role of various pathways, particularly the p38 MAPK and JNK signaling pathways, contributing to DUX4-driven muscle degeneration in FSHD. The MAP kinases are categorized into three primary families in mammals: ERKs (extracellular-signal-regulated kinases), JNKs (Jun amino-terminal kinases), and p38/SAPKs (stress-activated protein kinases) [40]. Given that p38 signaling can cause apoptosis, studies have investigated whether inhibiting this pathway could prevent DUX4-mediated cell death. In this context, losmapimod, a p38α/β inhibitor being evaluated in the aforementioned Phase 2b trial by Tawil et al. (NCT04003974), has shown promise in this regard (Table 1) [17].

In conjunction with studies of losmapimod, researchers have also focused on the JNK pathway. Notably, multiple peptides of the c-Jun (Jun) transcription factor, which serves as the substrate for JNK, were identified among the most phosphorylated peptides following DUX4 induction. The compound SP600125 has emerged as a candidate for further research due to its high selectivity for JNK and inhibiting phosphorylation of Jun; it exhibits approximately 20-fold selectivity over other kinases and a 300-fold selectivity compared to related MAPKs like ERK and p38 [41]. These specific therapeutic targets warrant further investigation to produce outcomes that can have a broader impact and ultimately improve the prognosis for individuals with FSHD.

#### 2.1.2. CRISPR-Cas9 and other gene editing technologies

Clustered regularly interspaced short palindromic repeats (CRISPR) and CRISPR-associated protein (Cas)9-mediated genome modification enable rapid and efficient editing of the genomes of various organisms, making it an increasingly popular tool for biological and therapeutic applications [42]. This technology offers a groundbreaking approach to address the underlying genetic cause of FSHD by directly targeting the D4Z4 repeat region responsible for DUX4 activation. Inherent to the widespread mechanism of CRISPR-Cas9 on the human genome, precise delivery to affected muscle tissues and concerns regarding off-target effects remain significant challenges. An additional challenge for FSHD is the repetitive nature of D4Z4 sequences; the presence of multiple CRISPR-Cas9 binding sites can lead to multiple DNA breaks along the locus [43]. This not only increases the risk of off-target effects, but also dilutes the editing efficiency at the intended site.

The first use of the CRISPR system in FSHD was reported in 2016, when Himeda et al. utilized an inhibitor fused to a “dead” Cas9 (dCas9) to target the DUX4 promoter in FSHD myoblasts. They distinguished two primary strategies for CRISPR-based DUX4 silencing: CRISPRe, which modifies genomic sequences using a functional Cas9, and CRISPRi, which utilizes an inactive (“dead”) Cas9 to inhibit gene expression without altering the DNA sequence. This CRISPRi approach effectively repressed DUX4 expression and its associated target genes at the D4Z4 locus [44]. Building upon these insights, a 2019 study by Goossens et al. further advanced the use of CRISPR technology by employing whole exome sequencing and CRISPR/Cas9 genome editing to repair a pathogenic SMCHD1 gene variant in patient-derived myoblasts, thereby providing a proof-of-concept for the feasibility of CRISPS/Cas9 as a targeted therapeutic strategy for FSHD [45].

In 2021, subsequent studies began to specifically address the off-target effects associated with CRISPR/Cas9 applications. Srikova et al. demonstrated a CRISPR/Cas9 editing strategy that utilized novel editing to modify the DUX4 locus while avoiding DNA double-strand breaks. They detected only around three sites that were edited in an off-target fashion, albeit to a much lesser extent than the intended target. This study illustrated a targeted approach with a high level of specificity, presenting a more efficient pathway to modulate gene expression without introducing excessive unwanted mutations [43]. Concurrently in 2021, Himeda et al. extended their 2016 usage of dCas9 by fusing it to epigenetic regulators to achieve stable repression of DUX4 expression with minimal off-target effects observed in FSHD myocytes, demonstrating its potential as a viable therapeutic option [46].

**Table 1.** Ongoing clinical trials for FSHD treatments as of November 2024.

Clinical trial registration number	Phase	Number of patients enrolled	Population	Trial period	Key clinical findings
**NCT05747924** [47]	2	76	18–65 years old FSHD patients	12 months	Improved muscle strength in both upper and lower limbs and reachable workspace when compared to the placebo.
**NCT05397470 (Losmapimod)** [48]	3	260	18–65 years old FSHD patients	48 weeks	Losmapimod failed to show an improvement in relative surface area (RSA), a measure of reachable workspace (RWS), versus placebo at week 48.
**NCT04264442 (Losmapimod)**	2	76	18–65 years old FSHD patients	48 weeks	No results posted
**NCT05548556 (RO7204239: humanized monoclonal antibody)**	2	48	18–65 years old FSHD patients	52 weeks	No results posted
**NCT06222827 (satralizumab: IL6-receptor antagonist)**	2	40	18–65 years old FSHD1 patients	96 weeks	No results posted
**NCT05747924 (AOC 1020)** [49]	1/2	100	16–70 years old FSHD patient	12 months	DUX4-regulated gene expression in muscle showed mean reductions >50% across multiple panels. All participants treated with AOC 1020 showed >20% reductions of DUX4-regulated genes. Participants showed ≥25% reductions in levels of novel circulating biomarkers and creatine kinase. Participants showed functional improvement trends, including increased upper and lower limb muscle strength and muscle function, compared to placebo and the ReSolve natural history study. Patient and clinician-reported outcomes showed trends of improvement.
**NCT06131983 (ARO-DUX4)**	1/2a	60	18–70 years old FSHD1 patients	Up to 360 days	No results posted

### 2.2. Antisense oligonucleotides (ASOs)

Antisense oligonucleotides (ASOs) represent a promising therapeutic approach for genetic disorders like FSHD by targeting the underlying genetic abnormalities responsible for the disease. ASOs are short, synthetic strands of nucleotides designed to bind specifically to messenger RNA (mRNA) transcripts, thereby modifying gene expression [50]. In FSHD, ASOs are engineered to target the toxic DUX4 mRNA, which is aberrantly expressed due to chromosomal deletions on 4q35 [51]. The DUX4 gene is typically silent in healthy individuals, but becomes inappropriately activated in muscle cells in patients with FSHD [52]. This inappropriate activation leads to muscle cell death and progressive muscular dystrophy. By binding to the DUX4 mRNA, ASOs can prevent its translation into the toxic DUX4 protein, thereby reducing its detrimental effects on muscle tissue. These ASOs work through various mechanisms, including promoting mRNA degradation via RNase H-mediated cleavage, sterically blocking the ribosome's ability to translate the mRNA, or modulating splicing patterns [53]. The ultimate goal of ASO therapy in FSHD is to reduce the production of the toxic DUX4 protein, thereby halting or slowing muscle degeneration. These findings have generated considerable interest in the potential of ASOs to become a disease-modifying treatment for FSHD, especially given the success of ASO therapies in other neuromuscular diseases, such as spinal muscular atrophy (SMA) and Duchenne muscular dystrophy (DMD) [54].

Preclinical studies have demonstrated the potential of ASOs to modulate DUX4 expression and mitigate muscle damage in FSHD models. In vitro studies using muscle cells derived from FSHD patients have shown that ASOs can effectively reduce DUX4 mRNA and protein levels, leading to improved cell survival and reduced cytotoxicity [55]. These promising findings have encouraged further investigation into ASO-based therapies for FSHD. In animal models, ASOs have been shown to suppress DUX4 expression and ameliorate muscle pathology [56]. Studies have indicated that local delivery of ASOs into affected muscles can significantly reduce DUX4 activity, leading to improvements in muscle function and a decrease in muscle inflammation [57]. These findings underscore the potential for ASOs to serve as a disease-modifying therapy for FSHD by targeting the root cause of the disorder. In another study, U7-small nuclear RNA (snRNA) antisense expression cassettes (U7-asDUX4) were developed to inhibit DUX4 expression in FSHD, offering a potential gene therapy approach that may require only a single administration and could be combined with other therapies to enhance DUX4 silencing [58].

Ongoing research aims to optimize the delivery methods of ASOs so that there is efficient distribution to the skeletal muscles, which are the primary tissues affected in FSHD [59]. Furthermore, researchers are focused on improving the stability and specificity of ASOs to minimize off-target effects and ensure long-term efficacy [60]. The ability to deliver ASOs systemically, rather than through localized injections, is also a critical area of development [57]. Initial studies have demonstrated that ASOs can effectively reduce DUX4 expression in vitro and in animal models, providing a foundation for further therapeutic exploration. While much work remains to be done, the initial research provides a strong rationale for pursuing ASOs as a treatment for this untreatable disease. If these early findings are translated successfully into clinical trials, ASO therapies could mark a significant advancement in FSHD treatment, offering hope for patients by directly addressing the molecular mechanism of their condition.

### 2.3. Myostatin inhibitors

Myostatin is a member of the TGF-β family of growth factors that acts as a negative regulator of muscle growth. Myostatin is a dimer that, in its latent form, is bound to its own inhibitory prodomain. When this prodomain is cleaved, the myostatin dimer becomes active and binds to a heterodimer receptor in the cell membrane, which after activation, leads to phosphorylation of Smad2/3, recruiting Smad 4 to form a Smad heterocomplex. This heterocomplex then reaches the nucleus, where it regulates transcription [61]. Due to its ability to control muscle growth, inhibiting myostatin offers a potential option in the treatment of muscle-wasting disorders [62]. Prior trials for myostatin inhibitors have displayed safety in use for patients with muscular dystrophies; however, they have not displayed significant clinical benefits to warrant active treatments. One phase II study in 2019, conducted by Acceleron Pharma, entailed the nonselective myostatin inhibitor, ACE-083 [63]. ACE-083 is a modified follistatin-Fc fusion protein that binds to myostatin [64]. In the first part of the study, the researchers examined 23 patients who received escalating doses of ACE-083 via intramuscular injection into either the biceps or tibialis anterior at three-week intervals for three months. This study demonstrated the safety profile of ACE-083, except for some injection site reactions, myalgias, and one incidence of lower extremity muscle swelling [65]. It was found that contractile muscle mass was increased by greater than 15% in the highest dose cohorts. The second part of the study was a randomized double-blind placebo-controlled study of the optimal dose, which was delivered intramuscularly to 56 patients at an interval of three weeks for six months [63]. The primary outcome measured for this part of the study was the change from the baseline of muscle volume, which would be measured via MRI. The results for this study are not publicly available; however, Acceleron has stated that the primary objective of increased muscle mass was achieved, but the measures of muscle function did not demonstrate clinical significance, ultimately terminating ACE-083 as a possible therapeutic option for FSHD treatment [66].

Before the Acceleron study, other myostatin inhibitors had been studied for their effect on muscular dystrophies. Wyeth Pharmaceuticals studied Stamulumab (MYO-029) in muscular dystrophies, establishing safety parameters, but were unable to establish significant improvement due to the study being underpowered [67]. Another study conducted by Acceleron examined the use of ACE-031, which is an ActRIIB-Fc fusion protein. ACE-031 demonstrated unacceptable side effects of gum and nose bleeds in addition to facial telangiectasias, leading to the termination of the trial [68]. Another trial conducted by Pfizer studied Domagrozumab, a neutralizing monoclonal antibody to myostatin, which had been shown to increase muscle mass in mice. Domagrozumab had been demonstrated to be safe and well tolerated in patients with DMD; however, it did not prove to be clinically effective, as demonstrated by there being no statistically significant difference in patients doing a four-stair climb between Domagrozumab and placebo [69]. While Domagrozumab did not clinically improve symptoms in patients with DMD, there may be potential for patients with FSHD, and additional studies may be required to determine its effectiveness.

An ongoing phase II trial by Hoffmann-La Roche is currently looking at the effects of RO7204239 against a placebo in people with FSHD. RO7204239 is an experimental anti-myostatin antibody that has been shown to block myostatin activity. The trial will examine the safety profile of RO7204239 compared with a placebo, utilizing MRI scans of thigh muscles after a 52-week course of treatment [70]. The results of this study can pave the way for additional studies to further assess the clinical effectiveness of RO7204239 and its possible use in the treatment of FSHD.

### 2.4. Cell-based therapies

Stem cell therapy is another potential avenue to explore in the context of FSHD treatment. The stem cells would be used to generate healthy myofibers with the DUX-4 gene inhibited or removed. These myofibers can then be used to replace diseased cells with healthy cells. Azzag et al. conducted a study utilizing pluripotent stem cells (PMC) in mouse models expressing DUX4. The results showed that transplanting healthy cells contributed to muscle regeneration and the implanted myofibers counteracted the DUX-4-induced fibrosis and led to increased muscle strength. These results indicate the potential of stem cell treatment for FSHD and emphasize the need for further studies examining the safety of PMC treatment in humans and clinical effectiveness in patients with FSHD [71].

It is important to consider the ethical considerations for stem cell treatment, such as its availability to patient populations. Treatments of this nature are likely to be costly and impose a source of financial burden upon patients. If this treatment modality is to be implemented, it would be worthwhile to find more effective ways to carry out this treatment, which can lower costs and save patients time. The current scope for stem cell therapy in the treatment of FSHD is limited and will need additional research to properly implement safely in humans.

## 3. Multidisciplinary management of FSHD

### 3.1. Role of occupational therapy

Occupational therapy (OT) plays a crucial role in supporting individuals with FSHD by addressing the multifaceted challenges they face in daily living [72]. OT enhances quality of life by focusing on activities of daily living (ADLs), instrumental activities of daily living (IADLs), health management, and social participation [73]. Patients with FSHD often experience pain and fatigue, which can significantly hinder their ability to perform essential tasks such as dressing, grooming, and preparing meals. Fatigue is especially pronounced in areas where muscle weakness is common, such as the shoulders and facial muscles, impacting communication and social interactions [74],[75].

To mitigate these challenges, occupational therapists frequently recommend home modifications to promote safety and accessibility. A mixed-methods survey of occupational therapists indicated that the most common modifications suggested include installing grab rails in showers and toilets, with over half of the therapists' clients implementing these recommendations. Additional adaptations, such as creating step-free showers, adding handrails at entrances, and removing shower screens, are also essential in facilitating independence and minimizing fall risks at home [76].

Moreover, advancements in assistive technology are paving the way for better support in daily activities. Studies on dynamic arm support devices for FSHD and other muscular dystrophies have shown that they can improve muscle coordination during daily activities [77],[78].

Along with physical therapy for shoulder strengthening, these devices help lift the arm, reduce muscle strain, and support greater independence [79]. While further research is needed, the future of OT in empowering FSHD patients is promising, with the potential to significantly improve their quality of life through personalized care and new assistive technologies.

### 3.2. Respiratory care and management

Although uncommon, advanced stages of FSHD can affect the muscles used in respiration. Respiration involvement symptoms include shortness of breath, daytime fatigue, morning headaches, and sleep disturbances [80]. A study done by Wohlgemuth et al. reported around 1% (10 patients) of the Dutch FSHD population were on nocturnal ventilatory support at home, with associated risk factors being wheelchair dependency, kyphoscoliosis, and severe muscle disease; nine patients in this study were reported to have a vital capacity of below 50% [81]. In a 10-year prospective study by Kilmer et al., around 50% of the 53 FSHD patient cohort had respiratory function tests consistent with restrictive lung disease, with 13% having severe respiratory dysfunction [82]. The need for respiratory care and management is apparent in cases such as these. Management can include invasive ventilation with intubation or non-invasive ventilation. These include continuous positive airway pressure (CPAP) or bi-level positive airway pressure (BiPAP) [83],[84].

## 4. Challenges and limitations in FSHD treatment

Due to the heterogeneity of presentations and the genetic complexity of pathogenesis, establishing a definitive and curative treatment path to FSHD has proven to be challenging, and as with other rare diseases, FSHD may more frequently result in slowed or misdiagnosis. The diversity in symptom severity and progression makes it difficult for clinical trials to adequately assess treatments due to the variable individual disease course [85]. Currently, there remains no single effective therapy for FSHD in lieu of a multidisciplinary approach, and while several pharmacologic trials have shown initial promise, they have yet to demonstrate sufficient treatment of the disease [14],[86]. Studying the use of gene therapy in modulating DUX4 in these patients has been complicated by the anomalous nature of DUX4 expression in the disease state and a limited number of natural mammalian models, without which human trials may pose difficulties [87],[88]. In addition, in vitro growth and differentiation of FSHD myoblasts are generally difficult, with low levels and high variability of DUX4 expression [6]. Genomic analysis of FSHD target regions using next-generation mapping and enrichment tools suffers from high costs, scarcity of protocols due to restrictions, dearth of infrastructure and expertise, and lack of testing in comprehensive studies [89]. As mentioned previously, antisense oligonucleotide therapeutics also show potential in the treatment of FSHD, but face challenges due to off-target effects, poor uptake in muscle tissue, and high expected cost if and when they reach the market [6]. Thus, more work is necessary to optimize clinical trial design in FSHD and fully elucidate the most promising pathomechanisms to target with disease-modifying therapies.

## 5. Future directions and conclusion

FSHD presents significant challenges due to its progressive nature, variability in clinical presentation, and the absence of disease-modifying treatments. This review highlights the diverse landscape of FSHD management, encompassing pharmacologic treatments, physical therapy, and exercise interventions, alongside the use of assistive devices and surgical strategies. Emerging therapeutic innovations, such as gene-targeted approaches, antisense oligonucleotides, myostatin inhibitors, and CRISPR-Cas9 technologies, represent promising avenues for addressing the genetic and molecular foundations of the disease. Future research in the treatment and management of FSHD should emphasize several key points to address the current therapeutic gaps and improve patient outcomes. Advancements in gene therapy, particularly in DUX4 silencing and CRISPR-Cas9 technologies, offer significant potential. However, continued research is needed to optimize delivery systems, minimize off-target effects, and ensure safety and efficacy in clinical settings. Additionally, given the genetic and phenotypic variability of FSHD, personalized treatment approaches are crucial. Identifying biomarkers that can predict disease progression and response to treatments will be instrumental in tailoring therapies to individual patients, allowing for more effective and patient-specific care. Improving the design of clinical trials is another priority, as current trials struggle with the variability in disease presentation and progression. Developing stratification methods that account for genetic and clinical differences will enhance the ability to assess therapeutic efficacy and accelerate the development of disease-modifying treatments. Moreover, the role of patient advocacy groups in advancing FSHD research cannot be understated. These groups are pivotal in securing funding, influencing regulatory pathways, and promoting awareness, all of which are vital for overcoming the regulatory and financial barriers that impede the development of novel therapies.

## Use of AI tools declaration

The authors declare they have not used Artificial Intelligence (AI) tools in the creation of this article.
